# Monitoring beam-quality constancy considering uncertainties associated with ionization chambers in Daily QA3 device

**DOI:** 10.1371/journal.pone.0246845

**Published:** 2021-02-17

**Authors:** Su Chul Han, Jihun Kim, Min Cheol Han, Kyung Hwan Chang, Kwangwoo Park, Ho Jin Kim, Dong Wook Kim, Jin Sung Kim

**Affiliations:** 1 Department of Radiation Oncology, Yonsei University College of Medicine, Seoul, Republic of Korea; 2 Department of Healthcare Solution, Douzone Bizon, Seoul, Republic of Korea; University of Texas MD Anderson Cancer Center, UNITED STATES

## Abstract

This study evaluates the changes occurring in the X-ray energy of a linear accelerator (LINAC) using a Daily QA3 detector system. This is accomplished by comparing the Daily QA3 results against those obtained using a water phantom. The X-energy levels of a LINAC were monitored over a duration of 1 month using the Daily QA3 system. Moreover, to account for the uncertainty, the reproducibility of the Daily QA3 ionization-chamber results was assessed by performing repeated measurements (12 per day). Subsequently, the energy-monitoring results were compared with the energy-change results calculated using the water-phantom percentage depth dose (PDD) ratio. As observed, the 6- and 10-MV beams experienced average daily energy-level changes of (-0.30 ± 0.32)% and (0.05 ± 0.38)%, respectively, during repeated measurements. The corresponding energy changes equaled (-0.30 ± 0.55)% and (-0.05 ± 0.48)%, respectively, when considering the measurement uncertainty. The Daily QA3 measurements performed at 6 MV demonstrated a variation of (2.15 ± 0.81)% (i.e., up to 3%). Meanwhile, the corresponding measurements performed using a water phantom demonstrated an increase in the PDD ratio from 0.577 to 0.580 (i.e., approximately 0.5%). At 10 MV, the energy variation in the Daily QA3 measurements equaled (-0.41 ± 0.82)% (i.e., within 1.5%), whereas the corresponding water phantom PDD ratio remained constant at 0.626. These results reveal that the Daily QA3 system can be used to monitor small energy changes occurring within radiotherapy machines. This demonstrates its potential for use as a secondary system for monitoring energy changes as part of the daily quality-assurance workflow.

## Introduction

Quality assurance (QA) is performed to that the machine used in clinical performs as expected. The QA radiotherapy machines is performed to confirm that the characteristics of the commissioned machines are identical to those of the tested prototype [1]. Periodic QA can be performed on a daily, weekly, monthly, or annual basis, and the machine status can be updated by comparing the measured and analyzed results against available guidelines. Guidelines for the specific acceptability criteria and tolerance levels used in the QA procedure have been suggested by the TG-40 and TG-142 task groups [2, 3]. Several studies have been performed to develop methods for the accurate and reliable evaluation of periodic measurements, and numerous vendors provide the required hardware and software systems. The users (medical physicists) develop and apply their own QA systems specific to their hospital and the systems employed therein. Alternatively, they might consider using vendor-supplied systems. In general, periodically deployed QA systems possess inherent measurement and setup uncertainties that must be reflected in their analysis results. As noted above, the daily QA has the highest frequency, and therefore, it can be used to evaluate radiotherapy machines prior to using them in practice.

The daily QA procedure involves mechanical inspection and a safety-related evaluation of the functional operational ability of the radiotherapy machine along with an evaluation of the X-energy constancy from a dosimetry perspective [2]. Several systems are used to perform daily QA in clinical radiotherapy [4–9]. These include the Daily QA3 system (Sun Nuclear Corporation; Melbourne, USA), which can be used to measure the dose output, flatness, symmetry, energy and radiation output, and light-field size. Using the Daily QA3 system, the daily QA results can be reviewed and assessed against the tolerance levels in the guidelines. Among the items measured by Daily QA3, monthly QA is recommended by TG-40 and TG-142 rather than daily QA for energy constancy (beam quality). Conventionally, energy constancy (i.e., beam quality) is evaluated by measuring the ratio of the percentage depth dose (PDD) at two different depths in a water phantom [2, 3]. Although this approach reveals the occurrence of significant changes in beam energy, its implementation for a multienergy machine is time-consuming with a low sensitivity to changes in beam energy [10].

Several approaches [5, 10–12] for evaluating beam quality via monitoring of changes in the beam flatness have been proposed as alternatives to the water-phantom-based method. Further, these approaches have been demonstrated to be more sensitive compared to methods that evaluate the beam quality at two depths [10, 11, 13]. Moreover, compared to conventional methods, these approaches reduce the time required to monitor energy fluctuations in multienergy machines. The Daily QA3 system uses the change in flatness as a measure of the beam quality [14], and it can easily monitor energy fluctuations in multienergy machines as a part of the daily QA workflow. However, the analysis results obtained using the Daily QA system reflect the operating condition of the radiotherapy machine being analyzed, thereby influencing the operator’s decision regarding its usage. Thus, it is important for users to understand the energy-measurement approach employed in the Daily QA3 system as well as its associated uncertainty with respect to energy change. Further, users must be aware of its sensitivity compared to that of conventional measurement methods.

In this study, the X-energy levels produced by a radiotherapy machine were monitored using the Daily QA3 system over a duration of 1 month. The observed uncertainties in the results were evaluated to assess the reproducibility of the Daily QA3 measurements. Subsequently, the Daily QA3 energy-monitoring results were compared with the energy (beam-quality) changes observed using the water phantom. The main objective of this study is to measure the extent to which the Daily QA3 system is sensitive to energy fluctuations within X-energy beams. The results obtained in this study accomplish this objective.

## Methodology

### Irradiation condition of Daily QA3 and monitoring of changes in X-energy

**Fig 1:**
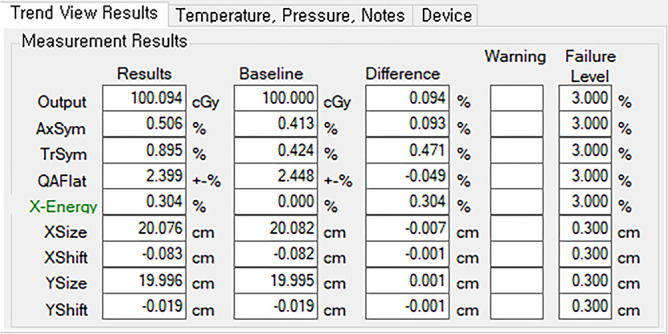
LINAC results obtained using Daily QA3 system viewed on Atlas QA graphic user interface.

All the measurements were performed using an Elekta Infinity linear accelerator (LINAC) (Elekta, Stockholm, Sweden) at two X-energy levels (6 and 10 MV). In Daily QA3, 13 ionization chambers (ICs) can be inserted to monitor the output, flatness, symmetry, and energy of radiotherapy machine beams. In this study, 12 diodes were used to measure and evaluate the size of the irradiation field [14]. The results measured by Daily QA3 are transferred to the ATLAS QA server and can be monitored via the network at any location. To perform daily QA using Daily QA3, a field size of 20 cm × 20 cm was delivered at a source-to-surface distance of 100 cm, resulting in the delivery of 100 MU. The PDD ratios (PDD_20_/PDD_10_) for X-energy and output were then calibrated using a water phantom and the Daily QA3 baseline was redetermined based on the water phantom results. The observed trend in the relative energy change measured by the Daily QA3 system was monitored using the Atlas QA program (illustrated in Fig 1).

The Daily QA3 manual prescribes the following relationship to calculate the relative energy change ($\bar{\Delta E}$) for a given flatness.

$$\mathrm{Flatness}\;(\%)=\mathrm{slope}\times \bar{\Delta E}+\mathrm{intercept}$$ (1)

In Eq (1), the slope is constant and has a vendor-provided value; when ΔE is zero, the intercept corresponds to the flatness value [14]. The manual provides a further definition of the flatness [14] expressed as

$$\mathrm{Flatness}\;(\%)=\frac{\mathrm{Average}\;\mathrm{Corner}\;\mathrm{IC}\;\mathrm{signal}}{\mathrm{Center}\;\mathrm{IC}\;\mathrm{signal}}\times 100,$$ (2)

where the numerator and denominator of the fraction denote the average of the signals measured at the four curved ICs located at the device corners and the signal measured in the IC located at the center of the Daily QA3 system, respectively. Both the signals are measured daily. In this study, $\bar{\Delta E}\pm \delta \bar{\Delta E}$ was defined in terms of the average and standard deviation of the relative energy changes measured using Daily QA3 over the course of the monitoring period (January 5–February 15, 2020). During this time, the X-energy of the radiotherapy machine was measured 28 times for daily QA.

### Calculation of X-energy changes via repeated measurements considering Daily QA3 uncertainty

**Fig 2:**
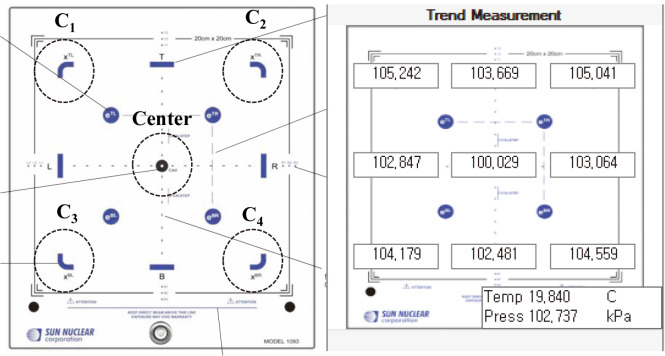
Atlas QA GUI showing locations and raw signals produced by ICs in Daily QA3 system.

Fig 1 depicts the graphic user interface (GUI) of the Atlas QA program, which users can use to monitor the energy changes recorded by the Daily QA3 system. More specifically, users can directly monitor signals from each of the four curved ICs located at the device corners as well as the parallel-plate IC located at the center of the device (illustrated in Fig 2).

To assess the uncertainty in the energy monitoring measurements obtained by Daily QA3, in this study, we evaluated the reproducibility of the five IC signals used for measuring energy change through repeated measurements. To evaluate the reproducibility of the Daily QA3 measurements, the baseline was newly determined and measured 12 times per day. And it was analyzed using a CV (coefficient of variation) to evaluate the reproducibility of the five ICs inserted into the Daily QA3

To evaluate the reproducibility of repeated measurements of energy change, given the uncertainties in the measurements produced by the five Daily QA3 ICs, we adapted Eq 2 to define the average of the flatness variation ($\bar{f(\%)}$) in consideration of the error propagati for each IC:

$$\begin{array}{l} \bar{f(\%)}=\frac{\mathrm{Average}\;\mathrm{Corner}\;\mathrm{IC}\;\mathrm{signal}\;(\bar{c)}}{\mathrm{Center}\;\mathrm{IC}\;\mathrm{signal}\;\left(C_{\mathrm{center}}\right)}=\frac{\bar{x}\pm \delta \bar{x}}{x_{\mathrm{center}}\pm \delta x_{\mathrm{center}}}\times 100\\ \;=\frac{\bar{x}}{x_{\mathrm{center}}}\pm \frac{\bar{x}}{x_{\mathrm{center}}}\sqrt{{\left(\frac{\delta \bar{x}}{\bar{x}}\right)}^{2}+{\left(\frac{\delta x_{\mathrm{center}}}{x_{\mathrm{center}}}\right)}^{2}}\times 100, \end{array}$$ (3)

where C_center_ denotes the sum of the average (x_center_) and standard-deviation (δx_center_) values of the signals obtained from the central IC via repeated measurements performed on each day. The value of $\bar{c}$ can be obtained from the average (*x*_*i*_) and standard-deviation (δ*x*_*i*_) values of each set of repeated corner IC signals as follows.

$$\begin{array}{l} \mathrm{Average}\;\mathrm{Corner}\;\mathrm{IC}\;\mathrm{signal}\;(\bar{c})=\frac{1}{n}\sum_{i=1}^{n}x_{i}\pm \delta x_{i}\;(n=4)\\ \;=\frac{1}{4}\left(x_{1}+x_{2}+x_{3}+x_{4}\right)\pm \frac{1}{4}\sqrt{\delta x_{1}^{2}+\delta x_{2}^{2}+\delta x_{3}^{2}+\delta x_{4}^{2}}\\ \;=\bar{x}\pm \delta \bar{x} \end{array}$$ (4)

Using the value of $\bar{f(\%)}$ determined via repeated measurements, $\bar{\Delta E}$ can be evaluated as

$$\bar{\Delta E}\pm u^{*}=\frac{\bar{f(\%)}+\mathrm{intercept}}{\mathrm{slope}},$$ (5)

where $\bar{\Delta E}$ ± u* denotes the average energy change, given the uncertainties within the Daily QA3 IC measurements. In Eq (5), the slope is constant, and the intercept corresponds to the flatness value when ΔE equals zero.

**Fig 3:**
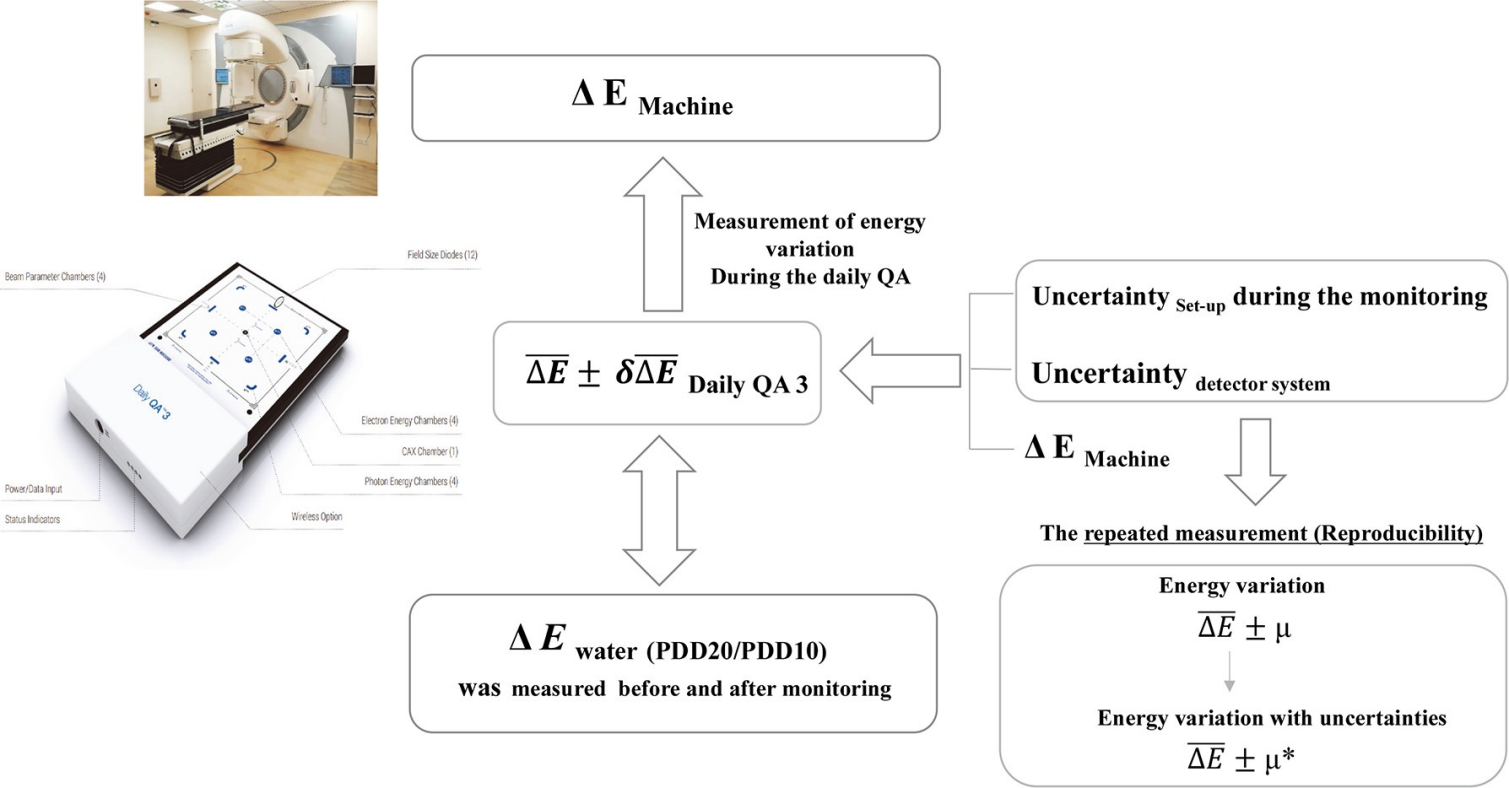
Overall concept for monitoring X-energy variation using Daily QA3 system.

Fig 3 depicts the concept applied in this study. As already stated, in this study, energy changes occurring in the radiotherapy machine were monitored for a month. The measurement results were influenced by the following factors—(1) variations in the setup (uncertainty _set-up_) of the Daily QA3 system during the monitoring period; (2) uncertainties within the Daily QA3 system with regard to energy-change monitoring (uncertainty _detector system_); and (3) changes in the energy levels produced by the radiotherapy machine (${\bar{\Delta E}}_{machine}$). The effects of the first and second factors were minimized by having the Daily QA3 system setup cross-verified by qualified medical professionals (QMPs) throughout the monitoring duration. Further, energy changes were repeatedly measured under identical irradiation conditions.

### Analysis of beam quality using water phantom

In this study, before monitoring the X-energy changes using the Daily QA3 system, the PDD ratio of the X-energy was calibrated using a water phantom having a field size of 10 cm × 10 cm and at an SSD of 100 cm. A farmer-type chamber (PTW; TN30013) was used to perform the beam-quality measurements. The PDD ratio was repeatedly measured by two QMPs before and after the Daily QA3 monitoring, and the results obtained were compared with the energy changes recorded by the Daily QA3 system via the trend analysis performed over the monitoring period.

## Results

### Monitoring energy changes using Daily QA3

**Fig 4:**
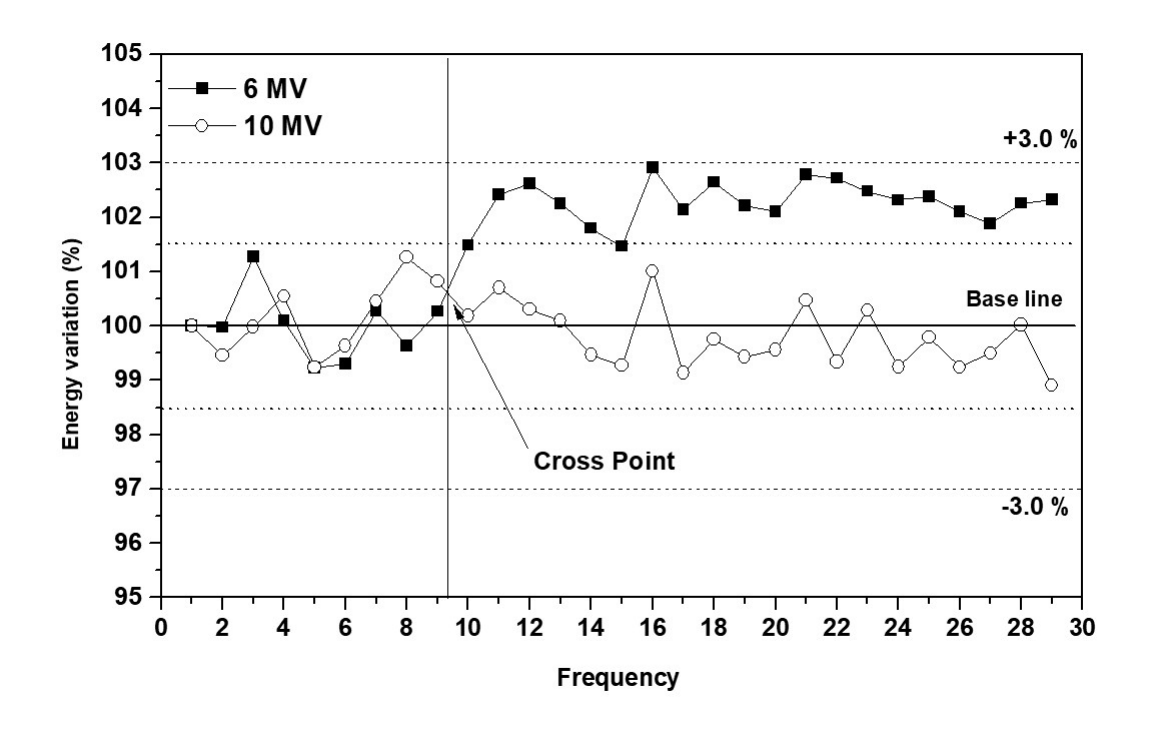
X-energy variation measured using Daily QA3 over the monitoring period ("cross point" indicates transition of the 6-MV energy trend).

Fig 4 depicts day-to-day variations in the measured X-energy values using Daily QA3. As observed, the energy changes at 10 MV are within ±1.5% of the baseline (100%) with an average energy change (${\bar{\Delta E}\pm \delta \bar{\Delta E}}_{10\;MV}$) of (-0.11 ± 0.62)%. In contrast, the energy change at 6 MV is within ±3% with a higher ${\bar{\Delta E}\pm \delta \bar{\Delta E}}_{6\;MV}$ of (1.56 ± 1.16)%.

During the monitoring period, the observed energy changes at 10 MV followed a trend similar to that of the changes observed at 6 MV up to the tenth measurement, beyond which the energy change at 6 MV demonstrated a gradual increase. Dividing the 6 MV changes into two groups yielded a ${\bar{\Delta E}\pm \delta \bar{\Delta E}}_{before\_6MV}$ value of (0.15 ± 0.74)% (n = 10) up to the tenth measurement, i.e., a value similar to ${\bar{\Delta E}\pm \delta \bar{\Delta E}}_{10\;MV}$ = (-0.11 ± 0.62)% (n = 29) over the entire period. Subsequent to the tenth measurement, ${\bar{\Delta E}\pm \delta \bar{\Delta E}}_{after\_6MV}$ equaled (2.30 ± 0.35)% (n = 19).

**Fig 5:**
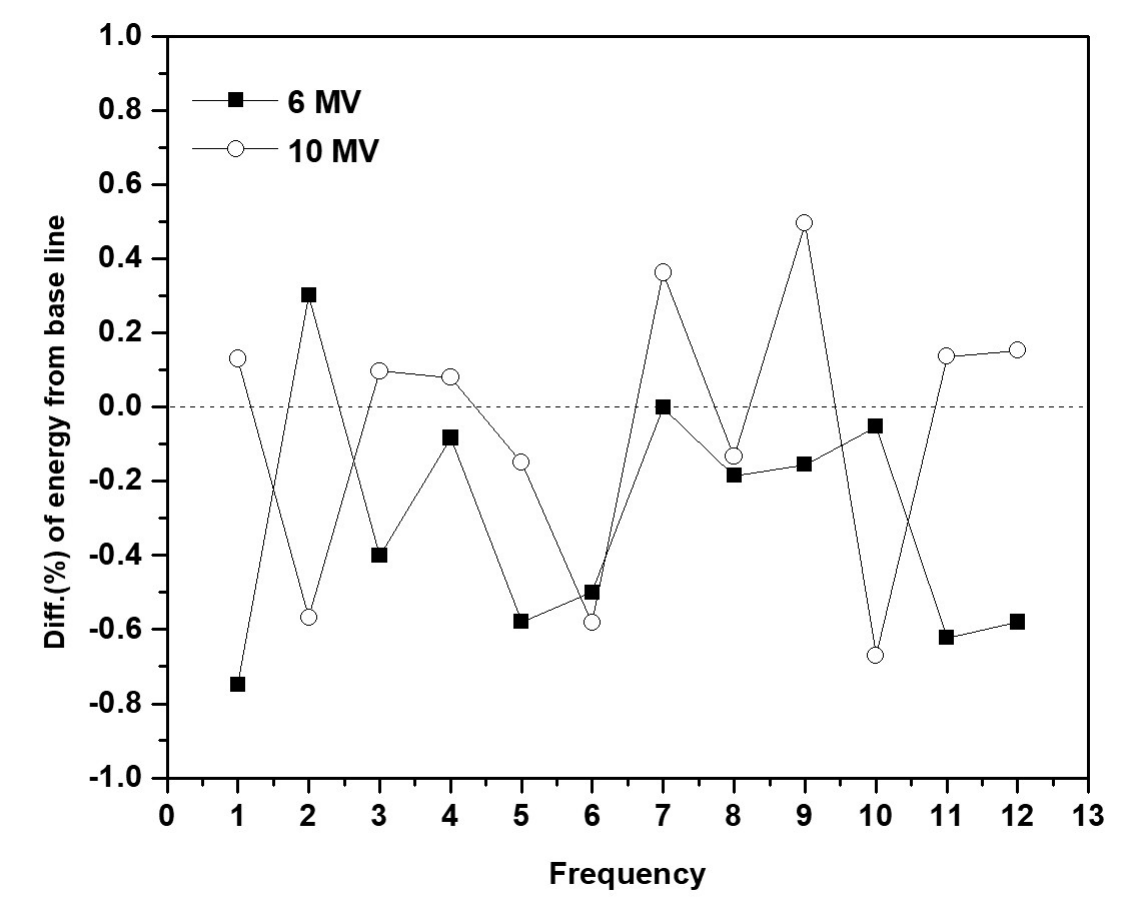
Variation of X-energy with energy level measured using Daily QA3 over the course of one day.

Fig 5 depicts the observed variations in relative energy against the baseline based on the beam energy measured over a single day. The average energy change at 6 MV (${\bar{\Delta E}\pm \delta \bar{\Delta E}}_{6MV}$) equaled (-0.30 ± 0.32)% (n = 12), whereas at 10 MV, $\left({\bar{\Delta E}\pm \delta \bar{\Delta E}}_{10\;MV}\right)$ it equaled (0.05 ± 0.38)% (n = 12). Repeated measurements of the energy changes occurring under identical conditions revealed the 10 MV beam to be less sensitive to changes in energy compared to the 6-MV beam, and the standard deviation ($\delta \bar{\Delta E}$) at both the energy levels remained within ±0.4%.

**Fig 6:**
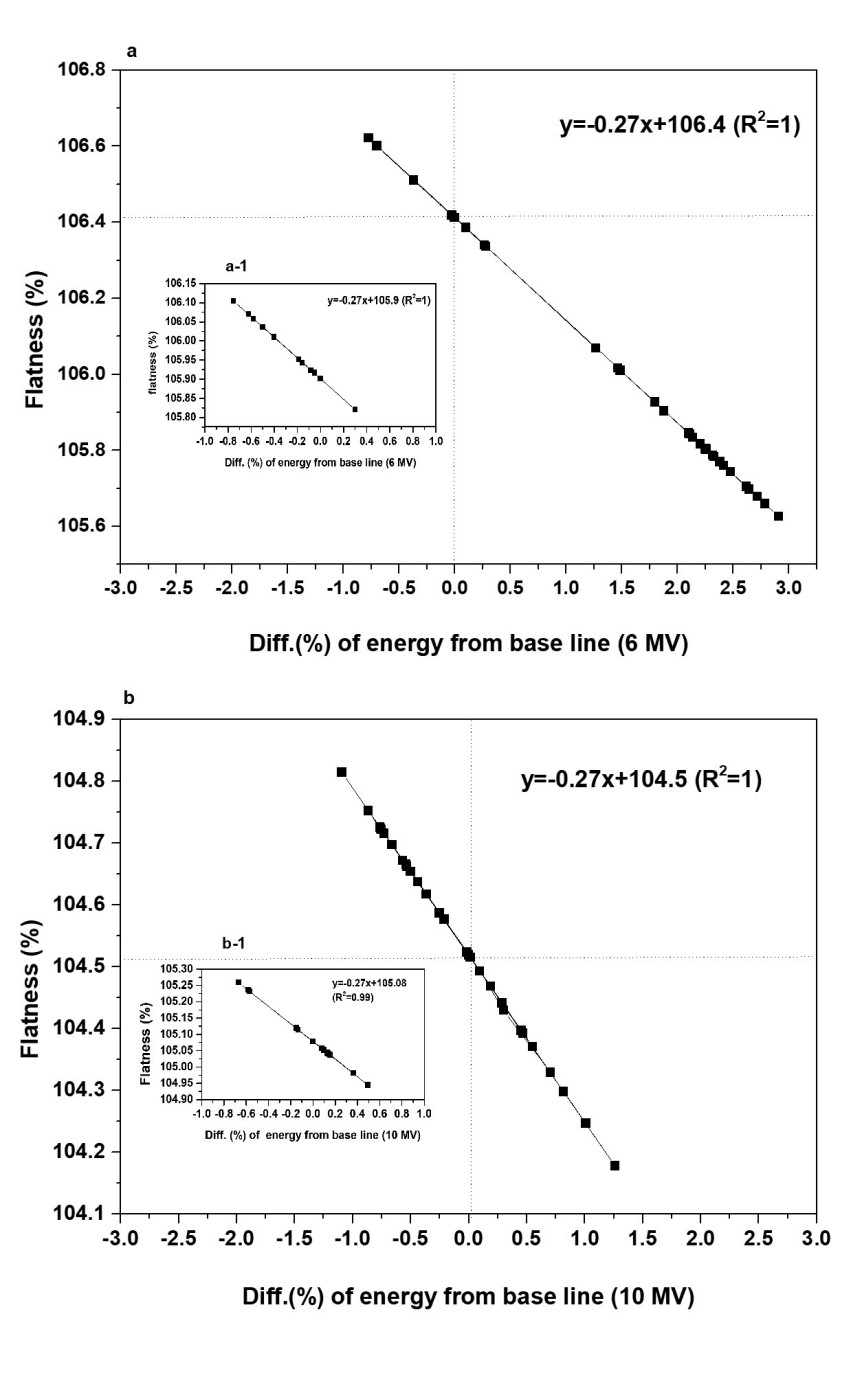
Correlations between energy change and flatness for (a) 6 MV and (b) 10 MV beams.

Fig 6(A) describes the correlation between the energy changes measured by the Daily QA3 system and the flatness (%) calculated from the IC signal using Eq (2). The relationship corresponding to the 6-MV beam over the entire monitoring period is given by Y = -0.27 X + 106.4 (R^2^ = 1). To evaluate the reproducibility of the monitoring results, the baseline was repeatedly adjusted, thereby modifying the above relationship to Y = -0.27 X + 105.9 (R^2^ = 1) (Fig 6(a-1)). Fig 6(B) depicts the result obtained for the 10-MV beam, for which the initial relationship Y = -0.27 X + 104.5 (R^2^ = 1) was obtained over the monitoring period. Through repeated measurements, this was subsequently corrected to Y = -0.27 X + 105.08 (R^2^ = 0.99) (Fig 6(b-1)). In both the cases, the adjustment of the baseline in the Daily QA3 system did not modify the slope, and the intercept Y_0_ could be determined using five raw IC signals obtained at the instant of baseline determination using Eq (1).

### Energy-change monitoring with uncertainties in five Daily QA3 IC signals

**Fig 7:**
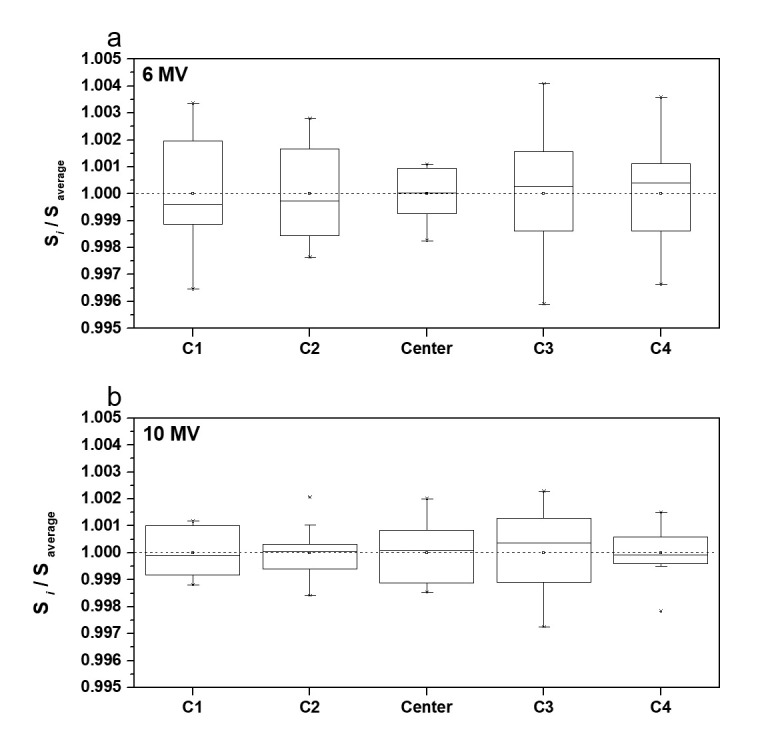
Changes in IC signals during repeated measurements performed in one day for (a) 6 MV and (b) 10 MV beams.

To evaluate the reproducibility of the results obtained using the Daily QA3 ICs, changes in each IC signal at both the energy levels (6 and 10 MV) were repeatedly monitored for a single day. The ratio of each signal (S_i_) to the average signal (S_average_) was calculated to evaluate the changes occurring in the individual IC signals over the measurement duration. For the 6 MV beam, the observed IC signal strengths demonstrated the distribution of C3 > C1 > C4 > Center with the largest change occurring in C3. Similarly, for the 10-MV beam, the IC signal in C3 experienced the largest change. However, the IC signals in this case were observed to be less sensitive compared to the case involving the 6 MV beam. Furthermore, all the signals demonstrated an X-energy variation range of ±0.5% (illustrated in Fig 7).

**Fig 8:**
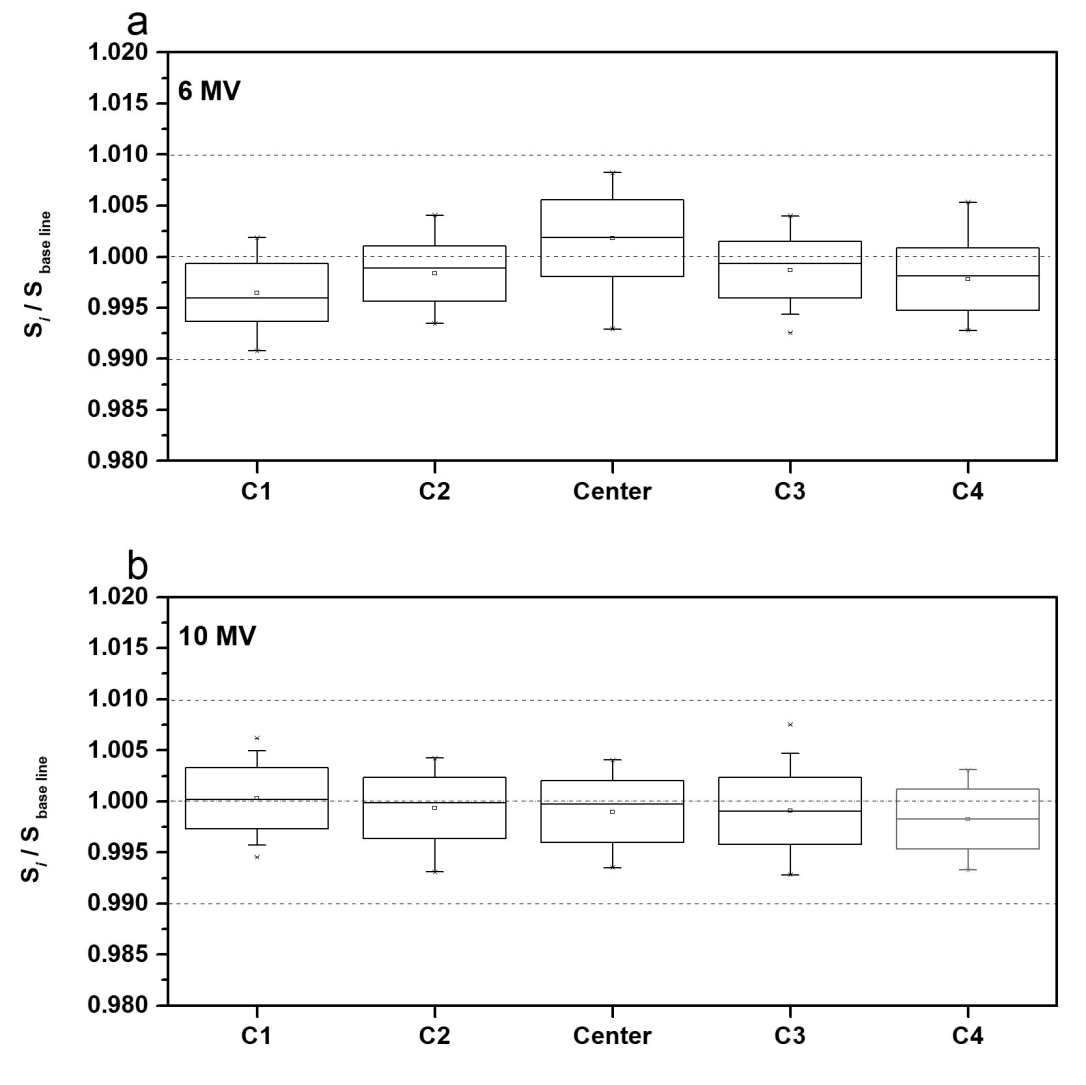
IC signal variations over the entire monitoring period for (a) 6 MV and (b) 10 MV beams.

Fig 8 depicts the changes in the signal energy produced by each IC signal over the entire monitoring period. To monitor these changes, the Daily QA3 system baseline was determined each day, and the ratio of each new signal (S_i_) to the newly determined baseline signal (*S*_*baseline*_) was re-evaluated. For both the beam energy levels, the observed changes in the IC signal remained within ±1%. Moreover, the variations in the central IC signal for the 6 MV beam exceeded those observed in other IC signals.

Further, in this study, the raw signals and $\bar{f(\%)}$ values of the IC signals were evaluated for both the beam-energy levels. Subsequently, the values of the average, standard deviation, and coefficient of variation (CV) of each IC output over the experiment duration were analyzed. The $\bar{f(\%)}$ values were calculated using Eqs (3) and (4) considering the uncertainty propagation for each IC raw signal. For the 6 and 10 MV beams, $\bar{f(\%)}$ equaled (105.97 ± 0.15)% and (105.09 ± 0.13)%, respectively. For all the IC signals obtained when the beam-energy level equaled 6 MV, the CV remained within 0.25%. For the 10 MV beam, all the CV remained within 0.15% (Table 1).

**Table 1:** Summary of IC signal changes during repeated measurements from an uncertainty perspective.

		Corner IC				C_center_	C_average_ $\left(\bar{c}\right)$	$\bar{f(\%)}$
		C_1_	C_2_	C_3_	C_4_			
6 MV	IC signal (*x*_*i*_±*δx*_*i*_)%	107.03 ±0.24	106.26 ±0.20	106.34 ±0.27	105.99 ±0.24	100.41 ±0.10	106.41 ±0.10	105.97 ±0.15
	CV (%)	0.22	0.19	0.25	0.22	0.09		
10 MV	IC signal (*x*_*i*_±*δx*_*i*_)%	105.67 ±0.09	105.01 ±0.26	105.40 ±0.15	105.68 ±0.16	100.37 ±0.12	105.48 ±0.06	105.09 ±0.13
	CV (%)	0.08	0.10	0.14	0.09	0.12		

Two methods were used to measure the energy variations obtained via repeated Daily QA3 measurements—(1) analysis of the average and standard deviation ($\bar{\Delta E}\pm \delta \bar{\Delta E}$) of the relative energy difference calculated from Atlas QA, as illustrated in Fig 1; (2) calculating the values of $\bar{\Delta E}$ ±u* with respect to the uncertainty for each raw IC signal via $\bar{f(\%)}$ (illustrated in Fig 2).

**Fig 9:**
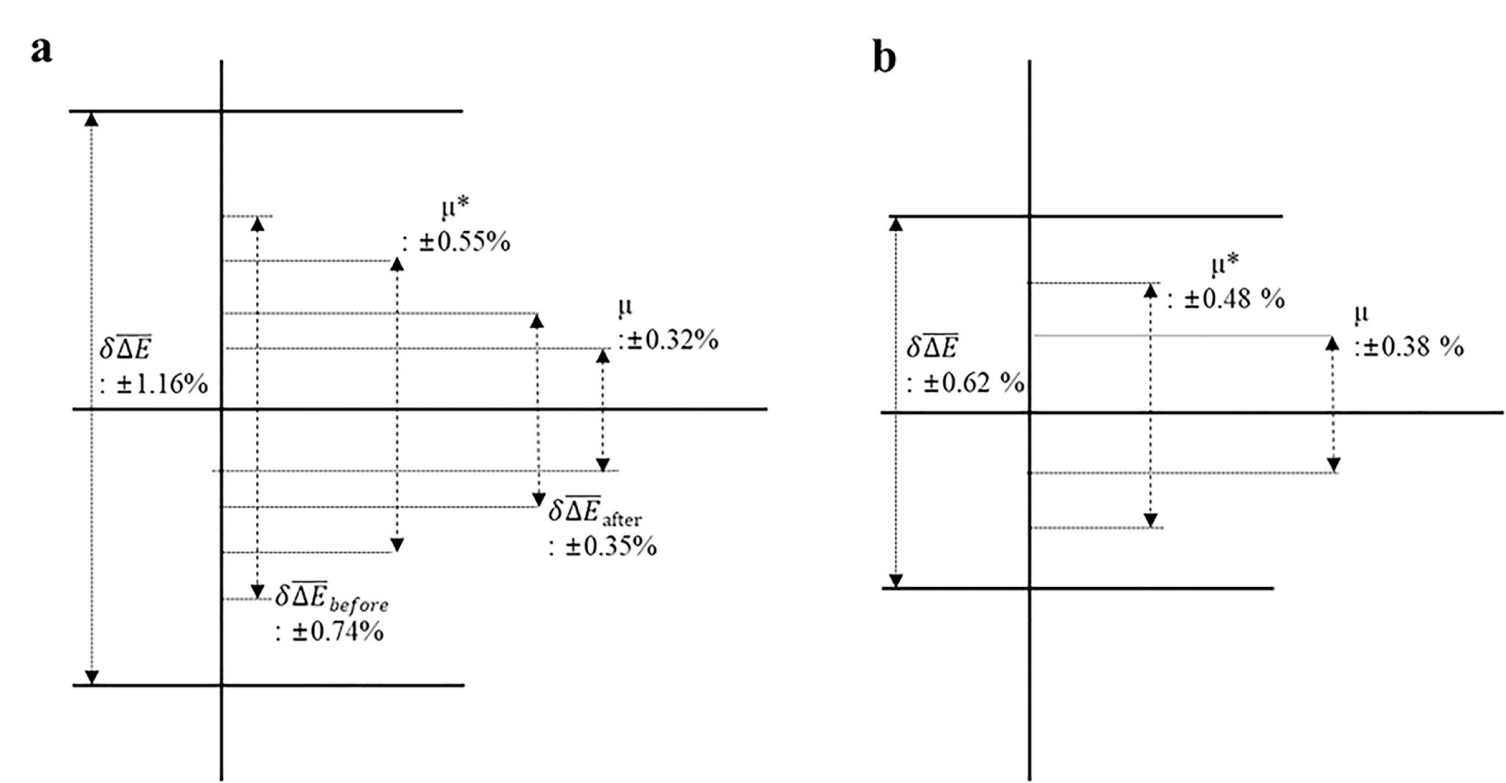
Uncertainties associated with monitored energy changes for (a) 6 MV and (b) 10 MV beams.

For the 6 MV beam, the values of ${\bar{\Delta E}\pm \delta \bar{\Delta E}}_{6MV}$ and $\bar{\Delta E}\pm u_{6MV}^{*}$ (which considers uncertainty propagation) equaled (-0.30 ± 0.32)% and (-0.30 ± 0.55)%, respectively. For the 10 MV beam, the corresponding values of ${\bar{\Delta E}\pm \delta \bar{\Delta E}}_{10MV}$ and $\bar{\Delta E}\pm u_{10MV}^{*}$ equaled (-0.05 ± 0.38)% and (-0.05 ± 0.48)%, respectively. As observed, at both the beam-energy levels, the application of the method considering the raw IC signal resulted in an increased uncertainty. Fig 9 depicts a comparison of these results with the energy-variation results obtained during the monitoring process.

### Post-QA3 beam-quality measurement using water phantom

In the Daily QA3 measurements performed for the 6 MV beam, the observed variation in the energy level demonstrated a gradual increase beyond the tenth measurement before stabilizing at a constant elevated value (Fig 4). Therefore, the variations in the measured energy values for the 6 MV beam were split into two groups at the tenth measurement interval. For comparison, the energy changes observed for the 10 MV beam were also divided into two groups comprising 10 measurement intervals each. The PDD ratios before and after monitoring were subsequently measured using a water phantom and compared with the Daily QA3 energies obtained for both the 6 and 10 MV beams. As observed, for the 6-MV beam, the PDD ratio before and after monitoring equaled 0.577 and 0.58, respectively (i.e., an increase of 0.003 or approximately 0.5%). For the 10 MV beam, the PDD ratio equaled 0.626 before and after monitoring. The average and standard-deviation values of the measurements are listed in Table 2.

**Table 2:** Comparison between changes in the energy measured using Daily QA3 system and water phantom PDD ratios.

Beam-energy level	Changes in energy	Daily QA3 ($\bar{\Delta E}\pm \delta \bar{\Delta E}$)%	PDD ratio (Average ±S.D)%
6 MV	Before 10^th^ interval	0.15 ± 0.74%	0.577 ± 0.0005
	After 10^th^ interval	2.30 ± 0.35%	0.580 ± 0.0005
	Difference (after -before)	2.15 ± 0.81%	0.003 ± 0.0007
10 MV	Before 10^th^ interval	0.18 ± 0.66%	0.626 ± 0.0005
	After 10^th^ interval	-0.24 ± 0.58%	0.626 ± 0.0005
	Difference (after -before)	-0.41 ± 0.88%	0.000 ± 0.0007

## Discussion and conclusions

According to the guidelines set forth by the TG-142 task group, energy constancy should not be evaluated on a daily but monthly basis instead [3]. Most daily measurement devices, however, could be used to measure X-energy constancy, and the QMPs can review the daily measurements based on the monthly QA results [15]. For such measurements, it is necessary for medical physicist to understand the mechanism of energy measurement in daily QA systems. This study evaluated the use of the commercial Daily QA3 system for daily energy measurements.

Several studies [1, 10, 12] have been performed to develop a robust method for energy-change monitoring using beam flatness as a governing parameter. There exists a reason for considering the changes in beam flatness for energy-change monitoring. The fixed shape and size of the flattering filter is optimized for specific beam energy. Therefore, changes in energy are inevitably correlated to the beam flatness. Accordingly, the proposed method is based on energy-change monitoring. Previous investigations were performed by artificially altering the beam energy using the bending magnet current (BMI) of the radiotherapy unit, and the correlation between the energy change and flatness was subsequently evaluated. Hossain et al. [12] evaluated the change in flatness by artificially changing the BMI in the range of ±20%. Their results revealed a linear relationship between the flatness change and BMI within a specific BMI range (-10–5% at a depth of 1.5 cm). In addition, the user manual supplied by the Daily QA3 manufacturer [14] states that the Daily QA3 system can be used to deduce the relationship between the energy change and flatness for beam energy levels in the range of 6 and 15 MV. Moreover, it is assumed that the artificial BMI change is equivalent to the change in X-energy. The relationship between change of energy and flatness is a linear relationship (R^2^ > 0.95) within ±3% of energy change. The relationships obtained in accordance with the operating manual were evaluated using five detectors located at depths of 1 cm each in the Daily QA3 system. The SSD was fixed at 100 cm. Binny et al. [16] inserted a physical wedge into the beam to artificially alter the flatness, and the resulting energy changes were evaluated using a Daily QA3 [16]. They determined the Daily QA3 system to have a tolerance of ±3%. The results reported by Hossain et al. [12] confirm that the change in flatness depends on the measurement depth. Therefore, in this study, the results of the flatness measurements performed repeatedly at the depth of 1 cm were compared with those performed at the depths of 5 and 10 cm under identical conditions. The measurements performed at the shallow (1 cm) depth were more sensitive than 5 cm and 10 cm depth. As observed, the difference between the measured flatness values as a function of the measurement depth was smaller in the case involving high beam energy (10 MV). The measured flatness at 10 cm depth equaled approximately 1 for both the 6 and 10 MV beams. This confirms that the five IC raw signals obtained from the Daily QA3 system are identical. The depth at which the flatness measurements are performed affects the value of the slope in Eq (1), and the relationship provided by the manufacturer has limited applicability depending on the measurement condition. Accordingly, it is important for medical physicist to understand the sensitivity of the flatness change when monitoring energy changes using the Daily QA3 system. Moreover, Gao et al. [1] proposed the off-axis ratio-based (OAR) metrics to be used in cases involving a flattening-filter-free (FFF) beam. However, the scope of this study is limited to monitoring energy changes in the 6 and 10 MV FF beams only using the Daily QA3 system. In future, we intend to evaluate the uncertainty associated with energy-change monitoring of FFF beams using the same system.

In their suggested correlation between the energy change and PDD ratio, Gao et al. [1] demonstrated that changing the BMI by ±10% alters the PDD ratio by ±1.5%. Hossain et al. [12] demonstrated that changing the BMI by 10% or more produces a change of 2% in the beam quality. Moreover, they confirmed that changes in the PDD ratio at 10 MV are less sensitive compared to those at 6 MV. Peng et al. [17] monitored the daily QA of an image-guided radiotherapy system using the Daily QA3. The Daily QA3 system has also been used to performed daily QA in proton therapy and photon radiotherapy [18, 19].

A major limitation of this study is that we did not artificially alter the beam energy using the BMI. Instead, the energy changes measured using the Daily QA3 system were monitored over a specific period, and post-monitoring changes in beam quality were evaluated using a water phantom.

As illustrated in Fig 6, the slopes of the respective equations remain constant irrespective of the beam-energy level owing to the use of the equation provided by the Daily QA3 manufacturer. Moreover, the Daily QA3 user manual [14] provides the results obtained when evaluating the flatness based on an induced artificial change in energy. In this case, the slopes of the equations corresponding to beam-energy levels of 6 MV and 10 MV equal -0.30 and -0.28, respectively. Additionally, the manual states that other values of the slope slightly exceeding -0.27 might be observed depending on the selected beam-energy level. To evaluate the dependence of the slope value on the observed energy change, we calculated the energy changes for different slope values. As observed, when the manufacturer’s suggested slope value of 0.27 was altered within a range of ±0.03 (i.e., from -0.24 to -0.30), the resulting average energy change lied in the range of (-0.025 ± 0.002)% to (2.926 ± 0.236)%. The corresponding error lies in the range of ±0.236%. The other variable in Eq (1) is the intercept (Y_0_). Its value is dependent on the baseline, the re-evaluation of which must only be considered after confirming the beam quality using the water phantom.

As described in Tables 1 and 2, despite the uncertainties associated with the respective IC signals, repeated reproducibility evaluations of the results obtained using the Daily QA3 system under fixed operating conditions revealed no significant differences in the energy-change standard deviations. This indicates that the observed differences in the reproducibility of the IC results have only a minor contribution toward the observed energy variation. For the 10 MV beam, the differences between the values of $\delta \bar{\Delta E}$ obtained during the monitoring period (0.62%) and the uncertainty reflecting values of u* (0.48) obtained during the repeated measurement process remained within 0.2%.

As illustrated in Fig 6, for the 6 MV beam, the monitored $\delta \bar{\Delta E}$ equaled 1.16%, or approximately twice the value of u* (0.55%), thereby reflecting the uncertainty associated with repeated measurements. This confirms that energy variations occurring in the 6 MV LINAC beam were detected by the Daily QA3 system, and the observed variations increased gradually after the tenth measurement. The splitting of the results of the 6 MV beam energy changes into two groups at the tenth measurement interval produced pre- and post-splitting standard deviations of ${\delta \bar{\Delta E}}_{before\_6MV}$ = 0.74% and ${\delta \bar{\Delta E}}_{after\_6MV}$ = 0.35%, respectively. Accordingly, the energy change ${\bar{\Delta E}}_{before\_6MV}$ demonstrates an increase of 1.5% relative to the baseline case. For the 6-MV beam, the observed differences between ${\delta \bar{\Delta E}}_{after\_6MV}\;(0.35\%)$, ${\delta \bar{\Delta E}}_{before\_6MV}\;(0.74\%)$, and u*(0.55%) remained within ±0.2% (Fig 9).

Considering the monthly PDD consistency stands at 2% [2], variations in the energy of a radiotherapy machine could be caused by several factors. Although such variations do not occur frequently in radiotherapy machines, they might affect the dose distribution delivered to a patient. Using a second measurement device, the QMPs can monitor the energy constancy as part of the daily QA procedure. However, it is necessary to understand the uncertainties associated with the measurement device.

The daily QA3 measurements performed in this study reveal that the energy of the 6 MV LINAC beam varies by 2.15 ± 0.81% (within 3%), whereas the corresponding water-phantom PDD ratio changes from 0.577 to 0.580—an increase of 0.003 (0.5%)—during the monitoring process. For the 10 MV beam, the corresponding PDD ratio (0.626 ± 0.0005) remains constant throughout the monitoring duration, whereas the Daily QA3 measurements reveal an energy variation of -0.41 ± 0.88% (within 1.5%). Overall, the findings of this study reveal the Daily QA3 system to have a small measurement uncertainty. Thus, the said system could be used for high-sensitivity monitoring of small energy changes in radiotherapy machines. This demonstrates the usefulness of the Daily QA3 as a secondary system for monitoring energy fluctuations in radiotherapy machines.

## Supporting information

S1 Data (XLSX)
